# Body Temperature Is Associated With Cognitive Performance in Older Adults With and Without Mild Cognitive Impairment: A Cross-sectional Analysis

**DOI:** 10.3389/fnagi.2021.585904

**Published:** 2021-02-12

**Authors:** Patrick Eggenberger, Michael Bürgisser, René M. Rossi, Simon Annaheim

**Affiliations:** ^1^Empa, Swiss Federal Laboratories for Materials Science and Technology, Laboratory for Biomimetic Membranes and Textiles, St. Gallen, Switzerland; ^2^Department of Health Sciences and Technology, Institute of Human Movement Sciences and Sport, ETH Zurich, Zurich, Switzerland

**Keywords:** mild cognitive impairment, dementia, Alzheimer’s disease, core body temperature, skin temperature, circadian temperature rhythm, cognitive performance, health monitoring

## Abstract

Wearable devices for remote and continuous health monitoring in older populations frequently include sensors for body temperature measurements (i.e., skin and core body temperatures). Healthy aging is associated with core body temperatures that are in the lower range of age-related normal values (36.3 ± 0.6°C, oral temperature), while patients with Alzheimer’s disease (AD) exhibit core body temperatures above normal values (up to 0.2°C). However, the relation of body temperature measures with neurocognitive health in older adults remains unknown. This study aimed to explore the association of body temperature with cognitive performance in older adults with and without mild cognitive impairment (MCI). Eighty community-dwelling older adults (≥65 years) participated, of which 54 participants were cognitively healthy and 26 participants met the criteria for MCI. Skin temperatures at the rib cage and the scapula were measured in the laboratory (single-point measurement) and neuropsychological tests were conducted to assess general cognitive performance, episodic memory, verbal fluency, executive function, and processing speed. In a subgroup (*n* = 15, nine healthy, six MCI), skin and core body temperatures were measured continuously during 12 h of habitual daily activities (long-term measurement). Spearman’s partial correlation analyses, controlled for age, revealed that lower median body temperature and higher peak-to-peak body temperature amplitude was associated with better general cognitive performance and with better performance in specific domains of cognition; [e.g., rib median skin temperature (single-point) vs. processing speed: *r_s_* = 0.33, *p* = 0.002; rib median skin temperature (long-term) vs. executive function: *r_s_* = 0.56, *p* = 0.023; and peak-to-peak core body temperature amplitude (long-term) vs. episodic memory: *r_s_* = 0.51, *p* = 0.032]. Additionally, cognitively healthy older adults showed lower median body temperature and higher peak-to-peak body temperature amplitude compared to older adults with MCI (e.g., rib median skin temperature, single-point: *p* = 0.035, *r* = 0.20). We conclude that both skin and core body temperature measures are potential early biomarkers of cognitive decline and preclinical symptoms of MCI/AD. It may therefore be promising to integrate body temperature measures into multi-parameter systems for the remote and continuous monitoring of neurocognitive health in older adults.

## Introduction

The aging population is steadily growing worldwide and age-related health issues and healthcare costs are concomitantly increasing. Therefore, novel approaches of remote and continuous health monitoring (telemonitoring) are becoming continuously more significant. The goal is to enable older adults to live at home independently for as long as possible and also provide older patients the opportunity to return home earlier after a stay in the hospital (Wagner et al., 2012). Remote and continuous health monitoring could allow detecting symptoms of illness and functional decline at the earliest possible stage. This facilitates the early implementation of preventive or therapeutic measures and thus potentially improves therapeutic efficacy (Pantelopoulos and Bourbakis, 2010). Together, early recognition and treatment may contribute to reducing healthcare costs for expensive inpatient monitoring and long-term treatment at hospitals or nursing homes for the elderly (Deen, 2015; Majumder et al., 2017). Recent advances in non-invasive sensor technologies and the mounting availability of wearable monitoring devices render the application of remote and continuous health monitoring increasingly feasible (Wang et al., 2017). It needs to be considered, however, that the accuracy of many of these devices has not yet been adequately validated (Dunn et al., 2018). Such monitoring systems typically assess physical activity patterns, indoor and outdoor position of the person, as well as important vital signs, including heart rate, respiratory rate, blood pressure, oxygen saturation, and body temperature (Pantelopoulos and Bourbakis, 2010; Wang et al., 2017). In home-care settings for older adults and in nursing homes for the elderly, core body temperature is commonly assessed with single-point measurements of oral or axillary temperature to detect acute infections and fever (High et al., 2009; Hamano and Tokuda, 2017). However, body temperature may additionally provide valuable information to monitor long-term overall health and neurocognitive health in older adults as subsequently described.

Healthy older persons’ core body temperatures were found to be in the lower range of age-related normal values and, consequently, lower core body temperature represents a biomarker for healthy aging and longevity (Flouris and Piantoni, 2015; Simonsick et al., 2016). In a 25-year follow-up study, older participants with core body temperatures in the lower 50% of the population, had significantly lower mortality than those with core body temperatures in the upper 50% (Roth et al., 2002; Waalen and Buxbaum, 2011). Furthermore, lower early morning core body temperature (assessed by oral thermometer measurement) is related to better physical fitness, including faster gait speed, reduced 400-m-walk time, and lower perceived exertion after walking 5 min at 0.67 m/s (Simonsick et al., 2016). Core body temperatures vary depending on the measurement site, with rectal temperatures being 0.3°C higher than ear-based, 0.7°C higher than oral, and 0.9°C higher than axillary temperatures (Lu et al., 2010). Older adults’ normal average core body temperature is 37.1°C (range across studies: 37.0–37.2°C) for rectal temperature measurement (Lu et al., 2010) and 36.3 ± 0.6°C for oral temperature measurement (Waalen and Buxbaum, 2011), respectively. In comparison, in younger people, the average rectal temperature is about 0.4°C higher (37.5°C) and the oral temperature is higher for about 0.7°C (37.0°C; Lu et al., 2010; Blatteis, 2012). This difference is caused by age-related decrements in vasomotor sweating function, skeletal muscle response, temperature perception, and physical behaviors which affect older adults’ ability to maintain optimal core body temperature (Lu et al., 2010).

In patients with Alzheimer’s disease (AD), core body temperature (assessed by rectal temperature measurement) is significantly elevated by 0.10°C on average (95% confidence interval 0.0068–0.1950°C) in comparison to normal values of non-demented older persons. This finding was reported in a meta-analysis summarizing six independent studies including a total of 90 AD patients and 78 healthy controls (Klegeris et al., 2007). The disturbance in core body temperature regulation represents a severe autonomic symptom of AD and might be an early indicator or predictor of AD onset (Carrettiero et al., 2015). The increased body temperature in AD pathology might be related to neuroinflammation (Klegeris et al., 2007). Pro-inflammatory cytokines, comprising interleukin (IL)-1, IL-6, and tumor necrosis factor (TNF)-α, are upregulated in the brain tissue and cerebrospinal fluid of patients affected by AD, which provides proof for brain inflammation (McGeer and McGeer, 2002; Calsolaro and Edison, 2016). These specific circulating cytokines are also believed to be the main endogenous pyrogens, inducing fever, and affecting thermoregulation (Netea et al., 2000). Nevertheless, whether neuroinflammation represents a protective or harmful mechanism in neurodegenerative diseases is still under debate (Calsolaro and Edison, 2016). Another hallmark of AD pathology (and other neurodegenerative disorders) that might be associated with the increased body temperature in affected individuals are intracellular inclusions of phosphorylated tau (p-tau) proteins in the brain (Carrettiero et al., 2015). Notably, hypothermia related to anesthesia in animal and human studies, significantly raises p-tau levels in the brain and cerebrospinal fluid, potentially impacting the genesis and/or progression of AD (Whittington et al., 2013). Within this context, it was speculated that the increased body temperature in AD patients may be the result of a compensatory reaction to counteract the elevated cytotoxic p-tau levels to preserve cognitive functioning (Carrettiero et al., 2015).

Continuous 24-h body temperature measurements depict the typical rise and fall of body temperature over a day and night, respectively, and reveal circadian rhythm disruptions in aging. These are common in older adults, however, are more severe in age-related neurodegenerative diseases, such as AD, AD-related dementias, and Parkinson’s disease (Leng et al., 2019). Dysfunctions of 24-h circadian rhythms encompass disturbed sleep-wake and rest-activity cycles, as well as impaired hormonal and body temperature rhythms, and manifest before symptoms of neurodegeneration are clinically evident (Leng et al., 2019). Therefore, parameters that reflect disruptions of circadian function may not only serve as early biomarkers of neurodegeneration but might also represent risk factors for the development of neurodegenerative diseases in healthy older adults (Ju et al., 2017; Leng et al., 2018; Musiek et al., 2018). The amplitude of the circadian core body temperature rhythm (assessed by rectal temperature measurement) is dampened by 20–40% in healthy older compared to younger adults, who typically show a lower temperature nadir (i.e., the lowest value during the circadian rhythm; Vitiello et al., 1986; Czeisler et al., 1992; Dijk et al., 2000; Hood and Amir, 2017). In patients with AD, circadian core body temperature amplitude (also assessed by rectal temperature measurement) was found to be reduced by even 50% compared to young adults, and this effect appeared to be more pronounced than in healthy older persons (Harper et al., 2005). Likewise, in patients with Lewy body dementia, the nocturnal amplitude of core body temperature (assessed by ingestible telemetric temperature pill measurement) was diminished in comparison to healthy controls (Raupach et al., 2019). Harper et al. (2005) proposed that the reduced core body temperature amplitude may be partially associated with lower levels of physical activity which are generally observable in both healthy older adults and patients with AD. Therefore, behavioral alterations related to normal aging in combination with disease-specific changes may drive disruptions of circadian rhythms in AD.

Certain early circadian rhythm disturbances associated with AD are already evident in older adults with mild cognitive impairments (MCI). These include a phase advance of the wrist skin temperature and motor activity circadian rhythm (i.e., a phase shift with minimum and maximum values occurring earlier during the day) compared to healthy persons of the same age (Ortiz-Tudela et al., 2014), as well as sleep disruptions based on polysomnography and melatonin measurements (Naismith et al., 2014). MCI is a condition referred to as a potential prodromal state of AD, where the individual shows objective cognitive impairments but is still able to independently perform activities of daily living (Langa and Levine, 2014; Vermunt et al., 2019). Consequently, these findings in MCI patients support the above-mentioned assumption that parameters assessing circadian rhythm, including body temperature measures among others, might serve as early biomarkers of preclinical and prodromal symptoms of AD. Nevertheless, to the best of our knowledge, it has not been investigated yet if single-point and long-term continuous body temperature measures are related to cognitive performance in older adults and if these parameters are different in older persons with or without MCI. This knowledge, however, would be critical to estimate the association of body temperature with neurocognitive health of the aging population and, consequently, to interpret body temperature data assessed with novel wearable systems for remote and continuous health monitoring.

Therefore, this study aimed to investigate the association of single-point and long-term continuous body temperature measures with cognitive performance in cognitively healthy older adults and in those with MCI. Thereby, this investigation will allow us to evaluate the suitability of various body temperature parameters as early biomarkers of cognitive decline, as well as preclinical and prodromal (i.e., MCI) symptoms of AD. We *hypothesized*, that: (1) lower single-point and long-term body temperature averages; and (2) higher long-term body temperature amplitudes are associated with better cognitive performance.

## Materials and Methods

### Study Design and Participants

The present study is a cross-sectional analysis that involves a single national center. Measurements were performed at Empa in St. Gallen, Switzerland. The ethics committee of eastern Switzerland approved the study protocol (project-ID 2019-01295, EKOS 19/105) and the study was registered at ClinicalTrials.gov with the identification code NCT04262674 (ClinicalTrials.gov., 2017). The planned methods remained unchanged after the start of the experiments and our reporting of this study adheres to the STROBE guidelines for observational studies (von Elm et al., 2014).

Healthy older adults and patients with MCI at the age of 65 years and above, living independently or in retirement homes for the elderly, were recruited from August until September 2019. Participants were recruited through local organizations that offer a variety of activities for older adults, including for instance lecture series or physical activities, as well as through advertisement in retirement homes, at primary care physicians, and in the organization of retired employees of our research institution. An information event was organized for interested persons. The measurement sessions were conducted from mid-September until October 2019. For eligibility, participants had to sign an informed consent and had to be able to walk at least 8 min at their preferred walking speed for measurements during activity, with or without walking aids. Older persons living in retirement homes and who are classified 0, 1, or 2 within the Swiss classification system for health-care requirements (BESA-levels, a German abbreviation for Bewohner-Einstufungs- und Abrechnungs-System) could participate in the study. Level 0 indicates that the person does not need care or treatment; level 1–2 indicates that the person only needs little care or treatment. Older adults with previously diagnosed dementia, e.g., Alzheimer’s disease, or recent head injury were excluded. In the case of acute or unstable chronic diseases (e.g., stroke, diabetes) and rapidly progressing or terminal illnesses, judgment by the participant’s primary care physician was required before inclusion in the study. Additional exclusion criteria for the subgroup of participants who agreed to ingest the telemetric temperature pill, comprised history of operations and/or disease related to the gastrointestinal tract within the last 5 years, implanted medical devices, planned MRI examination, as well as nausea, vomiting, constipation or abdominal pain within 1 month before the day of the planned measurement.

This cross-sectional analysis represents the baseline measurement of a subsequent randomized controlled trial (RCT) including several additional measures. Therefore, *a priori* power analysis (G*Power 3.1.3 Software; Faul et al., 2007) was calculated related to the requirements of the RCT. A total of 85 participants were needed to achieve 80% power for a two-group pre-and post-test design. The *α*-level was set at 0.05 and effect size *f* at 0.2, i.e., small to medium effect based on results of a similar study with older adults (Eggenberger et al., 2016).

### Experimental Protocol of Laboratory and Long-term Measurements

All participants attended two testing sessions in the laboratory at Empa in St. Gallen, Switzerland. The sessions lasted 1 h each and were performed on separate days. The *first test session* comprised measurements of skin temperatures at rest for 10 min in a seated position at controlled climatic conditions (22.1 ± 0.6°C, 46.9 ± 5.5% relative humidity). Participants were required not to talk and move during the measurement. Thereafter, measurements of skin temperatures were performed during activity, i.e., walking back and forth on a 20-m track at preferred speed for 8 min, under controlled climatic conditions (21.0 ± 0.3°C, 50.7 ± 4.8% relative humidity). These two test conditions were chosen to reflect potential body temperature differences among two typical daily activities. The *second test session* included the neuropsychological test battery to assess cognitive performance. Additionally, in a subgroup of the participants, long-term continuous skin and core body temperature measurements were conducted during the participants’ habitual daily activities. This measurement period lasted either from mid-evening until noon or from late evening until mid-afternoon of the next day, respectively, depending on when the participant was scheduled for the following test session in the laboratory. Due to the uniform periodicity of the circadian body temperature rhythm (i.e., the nadir in the early morning, peak in the late afternoon; Czeisler et al., 1999), the aforementioned slight variation of the measurement period timing would not affect the outcomes. Participants were blinded to the hypothesized study outcome; however, the investigators supervised and conducted the testing sessions, and blinding was therefore not applicable.

### Primary Outcome Measures

#### Body Temperature

**Figure 1:**
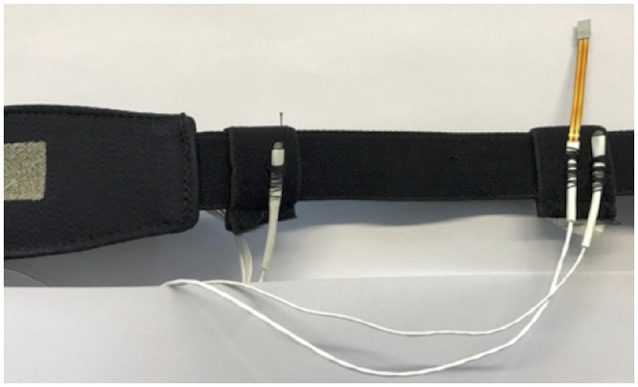
Skin temperature sensors (white) and heat flux sensor (orange) attached to the ECG belt. Notes: ECG and heat flux data were not analyzed in this study but were considered for another aspect of the project. ECG, electrocardiogram.

Skin temperatures (Ts) at the right lateral rib cage (ribs no. 5/6; Ts_rib_) and at the right scapula (inferior angle; Ts_scapula_) were measured using MSR thermistors (type DS18B20, MSR Electronics GmbH, Seuzach, Switzerland; 2 × 5 × 9 mm; polyolefin cover). Data were recorded at a 0.1 Hz sampling rate. Calibration of the temperature sensors (MSR thermistors) was performed at steady states between 15°C and 40°C (5°C intervals) with a calibration chamber (OptiCal, Michell Instruments, UK; Eggenberger et al., 2018). Temperature sensors were attached to a textile one-lead electrocardiogram (ECG) chest belt as illustrated in Figure 1 (Unico swiss tex GmbH, Alpnachstad, Switzerland; Fontana et al., 2019). ECG data were not analyzed in this study but were considered for another aspect of the project. We chose the two skin temperature measurement sites based on their previous application in a prediction model for core body temperature (Eggenberger et al., 2018) and compatibility with the described ECG chest belt.

In a subgroup of 19 participants, core body temperature was measured continuously every minute over 16 h using an ingestible telemetric temperature pill (Tc_pill_; e-Celsius Performance Pill, BodyCap, Hérouville Saint-Clair, France; weight: 1.7 g; size: 17.7 × 8.9 mm). Thereby, data was stored on the pill during the participants’ habitual daily activities and was retrieved upon return to the laboratory *via* a wireless transmission monitor (e-Viewer Performance monitor, BodyCap, Hérouville Saint-Clair, France) 16 h after ingestion, while the pill was not yet excreted by the body. The 16 h period was chosen based on the large inter-individual variability of transit times through the gastrointestinal tract (mean 27.4 h, range 4.6–82.8 h; Bongers et al., 2015). For data analyses, the first 4 h after ingestion of the telemetric pill were omitted as during this time the measurements may be affected by food or fluid intake as long as the pill has not transitioned into the intestinal tract (Wilkinson et al., 2008). Hence, the long-term continuous body temperature measurement comprised 12 h in total. Additionally, the subgroup participants were wearing the ECG chest belt with integrated skin temperature sensors for the same 16-h time period, to continuously assess long-term variations in skin temperatures (Ts_rib_, Ts_scapula_). Participants were instructed to follow their usual daily routines during the measurement period. None of them reported that they habitually pursue exceptionally prolonged or intense physical activities which could be deemed as atypical for this age-group.

### Secondary Outcome Measures

#### Cognitive Performance and Mild Cognitive Impairment

Seven neuropsychological tests were conducted to measure cognitive performance and to classify the participants as cognitively healthy or MCI. The test battery included the Quick Mild Cognitive Impairment screen (QMCI) to assess *general cognitive performance* (O’Caoimh et al., 2016), the Face-Name Associative Memory Exam (FNAME-12) for *episodic memory* (Papp et al., 2014), the category and letter fluency tests to assess* semantic and phonemic verbal fluency*, respectively (Clark et al., 2009; Mueller et al., 2015), the Trail Making Test B (TMT-B; Lezak et al., 2012) and the Stroop Word-Color Interference Test (Oswald and Fleischmann, 1997) representing the “shifting” and “inhibition” *executive functions*, respectively (Miyake et al., 2000), and the Trail Making Test-A (TMT-A; Lezak et al., 2012) for *processing speed*.

The following objective criteria were used to classify the participants as cognitively healthy or MCI for this study. Cognitively healthy was defined with a QMCI score ≥65, together with the other cognitive test scores differing by no more than 1.5 standard deviations (SD) from the group mean value. MCI was identified either if a QMCI score lower than 65 points was observed (O’Caoimh et al., 2016) or if one or more of the domain-specific cognitive test results were worse than 1.5 SD’s in relation to the group mean value (Petersen et al., 1999; Albert et al., 2011; Papp et al., 2020). The latter criterion and the exclusion of participants that were previously diagnosed with dementia correspond to the recommendations of the National Institute on Aging and the Alzheimer’s Association for diagnosing MCI (Albert et al., 2011). We did not include their further diagnostic criterion of subjective memory complaints as this was reported to increase misclassification rates (Edmonds et al., 2014). The QMCI is very accurate at distinguishing MCI from normal cognition (area under the curve = 0.90; O’Caoimh et al., 2016) and is also more accurate in this respect than other widely used short cognitive screening tests, including the Standardized Mini-Mental State Exam (SMMSE) and the Montreal Cognitive Assessment (MoCA; Glynn et al., 2019). For the MCI classification, the individual cognitive test scores were corrected for differences in age and education concerning normative data and reference studies (Tombaugh, 2004; Van der Elst et al., 2006; Papp et al., 2014; Romero et al., 2018).

### Data Processing

Calibration factors obtained from the calibration procedure mentioned above were applied to the skin temperature data. No filtering or smoothing of skin temperature data was performed. Long-term continuous temperature data from the ingestible telemetric pill (Tc_pill_) was processed with a one-dimensional fifth order median filter and a filter that excluded values that changed by more than 0.2°C to the median of the previous 1 min. MATLAB (version R2019a, MathWorks Inc., Natick, MA, USA) and Microsoft Excel 2016 software were used for data processing.

### Statistical Analyses

Independent *t*-tests were used to detect differences in demographic characteristics between the group including all participants and the subgroup, as well as differences in single-point body temperature and cognitive performance values between women and men. We calculated Spearman’s partial correlation coefficients to investigate relations between body temperature parameters and cognitive performance scores while controlling for age since this parameter is related to both body temperature (Waalen and Buxbaum, 2011) and cognition (Salthouse, 2019). Thereby, for the body temperature parameters assessed in the laboratory (single-point measurement), the median value during 10 min sitting and 8 min walking was used, respectively. For the body temperature parameters assessed during the 12-h continuous measurement, the median temperature and peak-to-peak (p–p) temperature amplitude (i.e., the range between minimum and maximum value) were calculated. Scatterplots of correlation analyses were visually inspected and *z*-scores were calculated to identify extreme outliers which were defined as *z*-scores >3.29 (i.e., values deviating more than 3.29 SD from the mean; Field, 2018). Thereby, it was assessed if outliers would unduly bias the results and, therefore, had to be excluded. Excluded values are reported in the results section. Furthermore, we performed Mann–Whitney tests to assess differences in single-point laboratory and 12-h continuous body temperature parameters among cognitively healthy and MCI participants. Missing values due to measurement error were not imputed and are also reported in the results section. Based on the use of preplanned hypotheses and the exploratory nature of this study, no adjustments of *p*-values were performed to account for possible multiple comparison effects. Although procedures to correct for multiple comparisons are discussed with controversy, many researchers in the field of biomedical sciences recommend not to apply them under the aforementioned premises (Perneger, 1998; Streiner and Norman, 2011; Armstrong, 2014; Ranstam, 2016). Instead, effect sizes (i.e., biological or clinical significance) and basic logic reasoning should represent the main criteria to interpret any statistical results (Cabin and Mitchell, 2000; Moran, 2003). Statistical analyses were computed with IBM SPSS Statistics software for Macintosh (version 26, IBM Corp., Armonk, NY, USA) with a significance level of *α* = 0.05. Considering that a negative result (i.e., *p* ≥ 0.05) is not proof of “no difference” since the *p*-value represents a continuous variable, we placed more emphasis on effect sizes and highlighted also results with notable moderate to large effects that did not achieve statistical significance (Halsey et al., 2015; Amrhein et al., 2017; Griffiths and Needleman, 2019). Magnitude of effect size *r* was considered as small for *r* = 0.10, moderate for *r* = 0.30 and large for *r* ≥ 0.50 (Cohen, 1988).

## Results

**Figure 2:**
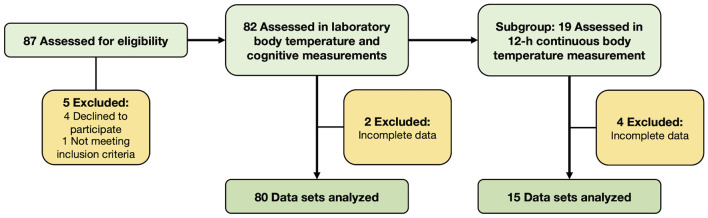
Participants’ flow and reasons for exclusion.

Eighty-seven participants initially enrolled in the study, out of which 82 persons were eligible for participation and completed the two experimental sessions in the laboratory (i.e., the single-point body temperature and cognitive performance measurements). The data of 80 participants was usable and was included in the analyses. Nineteen of these participants additionally took part in the 12-h continuous body temperature measurement during habitual daily activities and the data of 15 participants was used for the analyses. Participants’ flow and reasons for dropouts are depicted in Figure 2. The following values were missing due to measurement error in the data set from the 12-h continuous measurements of the analyzed 15 participants: all skin temperature values from one participant and the p–p amplitude values in Ts_rib_ and Ts_scapula_ from one other participant (both participants were included in the analyses as their Tc_pill_ data was valid). Demographic characteristics and mean values of single-point body temperature and cognitive performance are reported in Tables s 1, 2; respectively. The male participants were taller and heavier than the female participants, while the women performed better in the FNAME-12 and letter fluency cognitive tests. No other statistically significant differences were found within this context.

**Table 1:** Demographic characteristics.

Variable	All participants	Subgroup (12-h measurement)	*p*, two-tailed	Female	Male	*p*, two-tailed
*N*	80	15		50	30	
Sex, female	50,62.5%	8, 53.3%	0.509			
Age, years	74.6 (6.0)	75.2 (5.5)	0.702	74.0 (6.2)	75.5 (5.6)	0.297
Height, cm	166 (8)	167 (7)	0.772	162 (5)	174 (6)	<0.001*
Weight, kg	71.3 (12.6)	67.3 (12.4)	0.265	67.5 (12.0)	77.9 (10.8)	<0.001*
BMI, kg/m^2^	25.8 (4.3)	24.0 (3.3)	0.138	25.9 (4.8)	25.6 (3.3)	0.765
Habitation, independent/retirement home	77/3	15/0	0.461	48/2	29/1	0.851
Education, years	14.7 (2.1)	14.4 (2.0)	0.628	14.4 (2.1)	15.3 (2.0)	0.070
MCI	26, 32.5%	6, 40.0%	0.578	17, 34%	9, 30.0%	0.716
Hypertension	33, 41.3%	7, 46.7%	0.700	20, 40.0%	13, 43.3%	0.773
Diabetes mellitus	5, 6.3%	1, 6.7%	0.952	4, 8.0%	1, 3.3%	0.410
Depression, GDS score	1.9 (2.2)	2.3 (1.9)	0.438	2.2 (2.4)	1.4 (1.6)	0.096

Notes: data are numbers or means (standard deviation in brackets). Numbers for hypertension and diabetes mellitus are self-reported data from the health questionnaire. GDS scores >5 are normal, while scores >5 suggest depression (Sheikh and Yesavage, 1986). Bold values indicate statistical significance. BMI, body mass index; GDS, Geriatric Depression Scale; MCI, mild cognitive impairment; *p < 0.05, statistically significant.

**Table 2:** Single-point body temperature and cognitive performance values.

Variable	All participants	Female	Male	*p*, two-tailed
*N*	80	50	30	
Ts_rib_ sitting median,°C	34.5 (1.1)	34.4 (1.1)	34.6 (1.0)	0.619
Ts_rib_ walking median,°C	34.4 (1.0)	34.4 (1.0)	34.4 (1.0)	0.911
Ts_scapula_ sitting median,°C	33.4 (1.0)	33.3 (1.1)	33.4 (1.0)	0.583
Ts_scapula_ walking median,°C	33.4 (1.1)	33.4 (1.2)	33.5 (1.0)	0.640
QMCI, points	72.4 (11.6)	73.9 (12.3)	70.0 (10.1)	0.148
FNAME-12, points	52.7 (19.9)	58.0 (20.6)	43.8 (15.0)	0.001*
TMT-A, s	34.3 (10.5)	34.7 (11.2)	33.6 (9.3)	0.667
TMT-B, s	89.2 (53.3)	88.6 (42.6)	90.3 (68.3)	0.886
Stroop, s	44.6 (17.4)	42.2 (12.5)	48.7 (23.0)	0.107
Category fluency, number of words	21.8 (6.9)	22.6 (6.9)	20.4 (6.8)	0.157
Letter fluency, number of words	16.4 (5.0)	17.6 (5.0)	14.5 (4.3)	0.005*

Notes: data are means (standard deviation in brackets). *P*-values are for comparison of female and male participants. Bold values indicate statistical significance. FNAME-12, Face-Name Associative Memory Exam; QMCI, Quick Mild Cognitive Impairment screen; TMT-A, Trail Making Test part A; TMT-B, Trail Making Test part B; Ts_rib_, skin temperature at rib cage; Ts_scapula_, skin temperature at scapula; *p < 0.05, statistically significant.

### Correlation of Body Temperature Measures With Cognitive Performance

**Figure 3:**
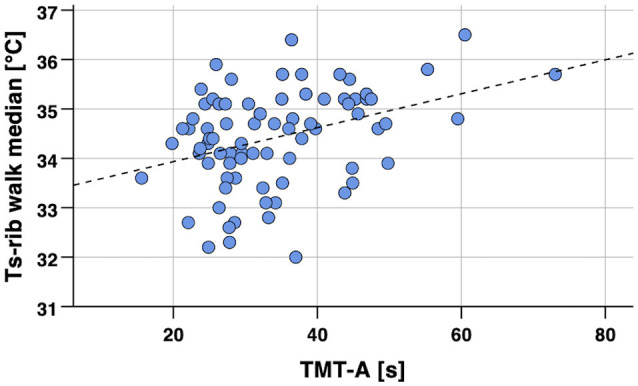
Scatterplot of skin temperature (Ts_rib_ median) during 8 min walking in the laboratory vs. cognitive performance (TMT-A, processing speed). Notes: Spearman’s partial correlation coefficient *r_s_* = 0.33, *p* < 0.002 (one-tailed, controlled for age). Regression line *y* = 33.25 + 0.03 × *x* (calculated from Pearson’s correlation analysis). TMT-A, Trail Making Test part A; Ts_rib_, skin temperature at the rib cage.

Spearman’s partial correlation analyses for the single-point body temperature parameters (median of Ts_rib_, Ts_scapula_) measured in the laboratory and the cognitive performance scores revealed statistically significant associations (*p* < 0.05) for Ts_rib_ during sitting and walking with TMT-A, while no other significant correlations were found as summarized in detail in Table 3. No differences in correlations with cognitive performance were obvious for the two body-temperature measurement conditions sitting or walking. Correlation analyses were conducted whilst controlling for age. An exemplary scatterplot with a significant correlation is presented in Figure 3. Upon visual inspection of the scatterplots and calculation of *z*-scores to screen for extreme outliers (defined as *z*-scores >3.29; Field, 2018), one value was excluded in order to remove undue bias (TMT-A = 168 s; *z-score* TMT-A = 7.25). Related to the above-mentioned significant difference between sexes in the FNAME-12 and letter fluency cognitive tests, we performed a supplementary Spearman’s partial correlation analyses (controlling for age) separately for the female and male participants. Thereby, we correlated the single-point body temperature parameters (median of Ts_rib_, Ts_scapula_) with these two cognitive tests. Within the male participants, significant correlations were evident for Ts_rib_ walking and Ts_scapula_ sitting with FNAME-12 (*r_s_* = –0.33, *p* = 0.041 and *r_s_* = –0.32, *p* = 0.046) and for Ts_rib_ sitting with letter fluency (*r_s_* = –0.32, *p* = 0.048), whereas no significant associations were found for the female participants.

**Table 3:** Spearman’s partial correlation analyses between single-point body temperature measurements and cognitive performance (controlled for age).

		Cognitive performance parameters													
Temperature parameters	Condition	QMCI (points)		FNAME-12 (points)		TMT-A (s)		TMT-B (s)		Stroop (s)		Category fluency (number of words)		Letter fluency (number of words)	
		*r_s_*	*p*	*r_s_*	*p*	*r_s_*	*p*	*r_s_*	*p*	*r_s_*	*p*	*r_s_*	*p*	*r_s_*	*p*
Ts_rib_ (°C)	Sitting	−0.16	0.084	−0.15	0.089	**0.28**	0.006*	0.06	0.298	0.11	0.158	0.11	0.166	−0.13	0.135
	Walking	−0.14	0.114	−0.15	0.098	**0.33**	0.002*	0.09	0.218	0.09	0.220	0.11	0.163	−0.05	0.319
Ts_scapula_ (°C)	Sitting	0.05	0.338	−0.05	0.319	0.12	0.145	−0.01	0.465	−0.03	0.401	0.17	0.072	−0.09	0.227
	Walking	0.03	0.409	−0.04	0.376	0.16	0.086	−0.01	0.475	−0.02	0.425	0.16	0.078	−0.02	0.433

Notes: for the temperature parameters, the median value during 10 min sitting and 8 min walking in the laboratory, respectively, was used for the analyses. *P*-values are one-tailed. Bold values indicate statistical significance. Due to the use of preplanned hypotheses and the exploratory nature of this study, *p*-values were not adjusted for possible multiple comparison effects (Perneger, 1998; Streiner and Norman, 2011; Armstrong, 2014; Ranstam, 2016). FNAME-12, Face-Name Associative Memory Exam; QMCI, Quick Mild Cognitive Impairment screen; TMT-A, Trail Making Test part A; TMT-B, Trail Making Test part B; Ts_rib_, skin temperature at rib cage; Ts_scapula_, skin temperature at scapula; *p < 0.05, statistically significant.

**Figure 4:**
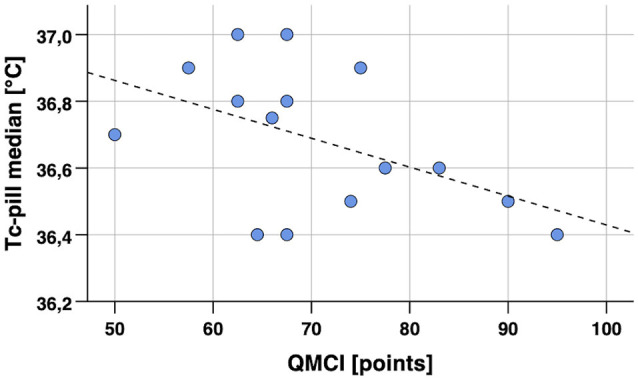
Scatterplot of core body temperature (Tc_pill_ median) in 12-h continuous measurement during habitual daily activities vs. cognitive performance (QMCI, general cognitive performance). Notes: Spearman’s partial correlation coefficient *r_s_* = –0.47, *p* = 0.045 (one-tailed, controlled for age). Regression line *y* = 37.30–0.0009 × *x* (calculated from Pearson’s correlation analysis). Tc_pill_, core body temperature from ingestible telemetric temperature pill.

**Figure 5:**
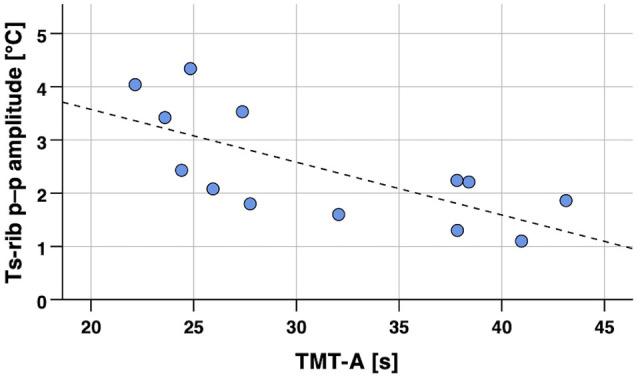
Scatterplot of skin temperature p–p amplitude (Ts_rib_) in 12-h continuous measurement during habitual daily activities vs. cognitive performance (TMT-A, processing speed). Notes: Spearman’s partial correlation coefficient *r_s_* = –0.59, *p* = 0.021 (one-tailed, controlled for age). Regression line *y* = 5.56–0.1 × *x* (calculated from Pearson’s correlation analysis). p–p, peak-to-peak; Ts_rib_, skin temperature at the rib cage.

Spearman’s partial correlation analyses for body temperature parameters measured during 12 h of habitual daily activities (median and p–p amplitude of Ts_rib_, Ts_scapula_, Tc_pill_) and cognitive performance scores resulted in statistically significant correlations (*p* < 0.05) for Ts_rib_ with TMT-A and Stroop, for Ts_scapula_ with TMT-A, and Tc_pill_ with QMCI, FNAME-12, category, and letter fluency. Additionally, notable non-significant results with moderate to large effect size were found for Ts_rib_ with QMCI and FNAME-12, for Ts_scapula_ with Stroop, and Tc_pill_ with QMCI and Stroop. Correlation analyses were again controlled for age. The corresponding correlation coefficients are reported in Table 4 and two exemplary scatterplots with significant correlations are depicted in Figures 4, 5. No extreme outliers (defined as *z*-scores >3.29; Field, 2018) were identified in this data set.

**Table 4:** Spearman’s partial correlation analyses between 12-h continuous body temperature measurements and cognitive performance (controlled for age).

		Cognitive performance parameters													
Temperature parameters	Value	QMCI (points)		FNAME-12 (points)		TMT-A (s)		TMT-B (s)		Stroop (s)		Category fluency (number of words)		Letter fluency (number of words)	
		*r_s_*	*p*	*r_s_*	*p*	*r_s_*	*p*	*r_s_*	*p*	*r_s_*	*p*	*r_s_*	*p*	*r_s_*	*p*
Ts_rib_ (°C)	Median	−0.04	0.450	0.13	0.332	−0.04	0.453	0.10	0.375	**0.56**	0.023*	−0.11	0.358	0.17	0.286
Ts_scapula_ (°C)		0.15	0.311	0.19	0.265	0.04	0.455	0.24	0.216	**0.46**	**0.055**	−0.18	0.277	0.03	0.457
Tc_pill_ (°C)		**−0.47**	0.045*	−0.21	0.234	0.22	0.221	−0.02	0.472	−0.18	0.272	**−0.53**	0.026*	−0.15	0.308
Ts_rib_ (°C)	p–p Amplitude	**0.38**	**0.112**	**0.49**	**0.054**	**−0.59**	0.021*	−0.07	0.417	0.21	0.259	−0.02	0.475	0.06	0.425
Ts_scapula_ (°C)		0.15	0.323	0.23	0.241	**−0.57**	0.028*	−0.11	0.365	−0.07	0.421	−0.25	0.222	−0.12	0.361
Tc_pill_ (°C)		**0.39**	**0.083**	**0.51**	0.032*	0.14	0.315	0.17	0.279	**0.38**	**0.088**	0.04	0.442	**0.48**	0.041*

Notes: temperature parameters were assessed during 12 h of habitual daily activities. *p*-values are one-tailed. Bold values indicate significant or notable non-significant results with moderate to large effect size. Due to the use of preplanned hypotheses and the exploratory nature of this study, *p*-values were not adjusted for possible multiple comparison effects (Perneger, 1998; Streiner and Norman, 2011; Armstrong, 2014; Ranstam, 2016). FNAME-12, Face-Name Associative Memory Exam; p–p, peak-to-peak; QMCI, Quick Mild Cognitive Impairment screen; Tc_pill_, core body temperature from ingestible telemetric temperature pill; TMT-A, Trail Making Test part A; TMT-B, Trail Making Test part B; Ts_rib_, skin temperature at rib cage; Ts_scapula_, skin temperature at scapula; *p < 0.05, statistically significant.

### Differences in Body Temperature Measures Between Cognitively Healthy and MCI Participants

Based on the cognitive performance assessments, 54 participants were classified as cognitively healthy and 26 participants met the criteria for MCI. Mann–Whitney tests showed statistically significant differences (*p* < 0.05) between cognitively healthy and MCI participants in the single-point body temperature parameters Ts_rib_ median during sitting and walking in the laboratory as presented in Table 5.

**Table 5:** Comparison of single-point body temperature measurements between cognitively healthy and MCI participants.

		Mean (SD)		Mann–Whitney test			
Temperature parameters	Condition	Healthy *n* = 54	MCI *n* = 26	*U*	*z*	*p*, one-tailed	*r*
Ts_rib_ (°C)	Sitting	34.3 (1.0)	34.8 (1.2)	867.5	1.70	0.045*	**0.19**
	Walking	34.3 (0.9)	34.7 (1.1)	879.0	1.82	0.035*	**0.20**
Ts_scapula_ (°C)	Sitting	33.3 (1.0)	33.5 (1.1)	783.5	0.84	0.201	0.09
	Walking	33.4 (1.0)	33.5 (1.3)	773.0	0.73	0.233	0.08

Notes: for the temperature parameters, the median value during 10 min sitting and 8 min walking in the laboratory, respectively, was used for the analyses. Bold values indicate statistical significance or notable non-significant results with moderate to large effect size. p–p, peak-to-peak; MCI, mild cognitive impairment; SD, standard deviation; Ts_rib_, skin temperature at rib cage; Ts_scapula_, skin temperature at scapula; *p < 0.05, statistically significant; *p < 0.05, statistically significant.

**Figure 6:**
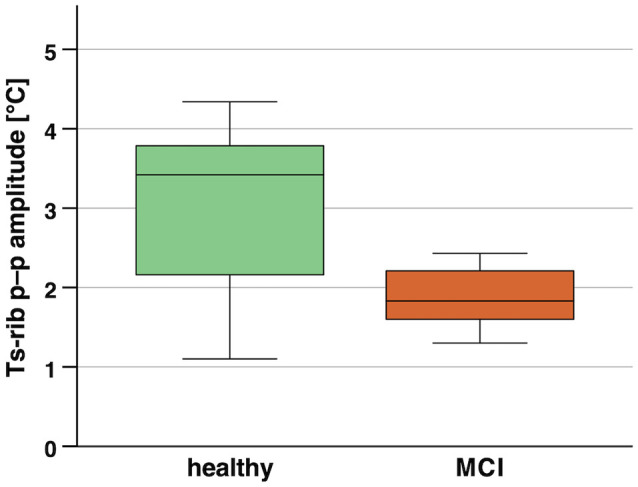
Boxplots of skin temperature p–p amplitude (Ts_rib_) in 12-h continuous measurement during habitual daily activities of healthy vs. MCI participants. Notes: *p* = 0.051 (not significant, one-tailed), *r* = –0.48 (large effect). MCI, mild cognitive impairment; Ts_rib_, skin temperature at the rib cage.

In the subgroup performing the additional long-term continuous body temperature measurement, nine participants were classified as cognitively healthy and six participants met the criteria for MCI. Mann–Whitney tests revealed no statistically significant difference (*p* ≥ 0.05) between cognitively healthy and MCI participants in the 12-h continuous body temperature parameters. Notable non-significant differences with moderate to large effect size were found for Ts_rib_ median and p–p amplitude, Ts_scap_ p–p amplitude, and Tc_pill_ p–p amplitude as shown in Table 6. Boxplots for the p–p amplitude of Ts_rib_ in healthy and MCI participants are illustrated in Figure 6.

**Table 6:** Comparison of 12-h body temperature measurements between cognitively healthy and MCI participants.

		Mean (SD)		Mann–Whitney test			
Temperature parameters	Value	Healthy *n* = 9	MCI *n* = 6	*U*	*z*	*p*, one-tailed	*r*
Ts_rib_ (°C)	Median	35.6 (0.5)	36.0 (0.4)	33.5	1.23	0.114	0.33
Ts_scapula_ (°C)		35.1 (0.6)	35.3 (0.5)	30.0	0.78	0.246	0.21
Tc_pill_ (°C)		36.7 (0.2)	36.7 (0.2)	31.0	0.48	0.345	0.12
Ts_rib_ (°C)	p–p Amplitude	3.0 (1.2)	1.9 (0.4)	9.0	−1.71	**0.051**	**−0.48**
Ts_scapula_ (°C)		3.3 (1.1)	2.4 (0.6)	10.0	−1.57	**0.069**	**−0.44**
Tc_pill_ (°C)		1.5 (0.6)	1.1 (0.2)	15.0	−1.43	**0.091**	**−0.37**

*Notes: temperature parameters were assessed during 12 h of habitual daily activities. Bold values indicate statistical significance (<0.05) or notable non-significant results with moderate to large effect size. p–p, peak-to-peak; MCI, mild cognitive impairment; SD, standard deviation; Tc_pill_, core body temperature from ingestible telemetric temperature pill; Ts_rib_, skin temperature at rib cage; Ts_scapula_, skin temperature at scapula*.

**Figure 7:**
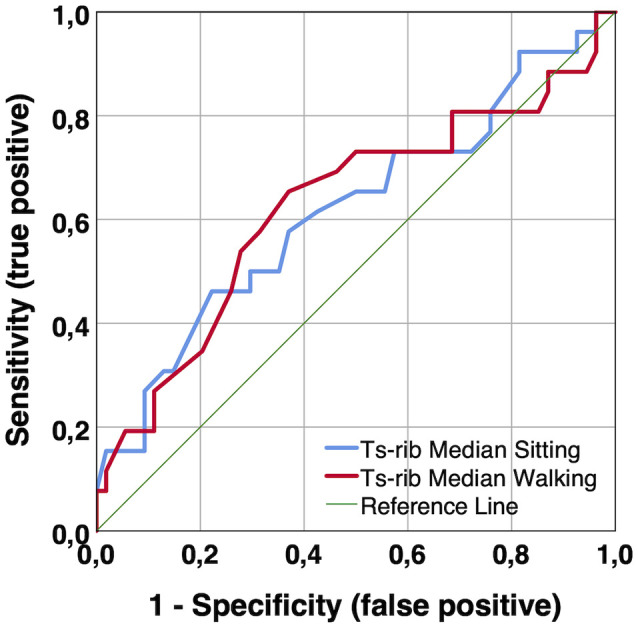
ROC curves of median skin temperatures (Ts_rib_) measured in the laboratory to predict MCI. Notes: Ts_rib_ median during sitting and walking, AUC = 0.618 (*p* = 0.089) and 0.626 (*p* = 0.069), respectively. AUC, area under curve; MCI, mild cognitive impairment; Ts_rib_, skin temperature at rib cage; ROC, receiver operating characteristic.

**Figure 8:**
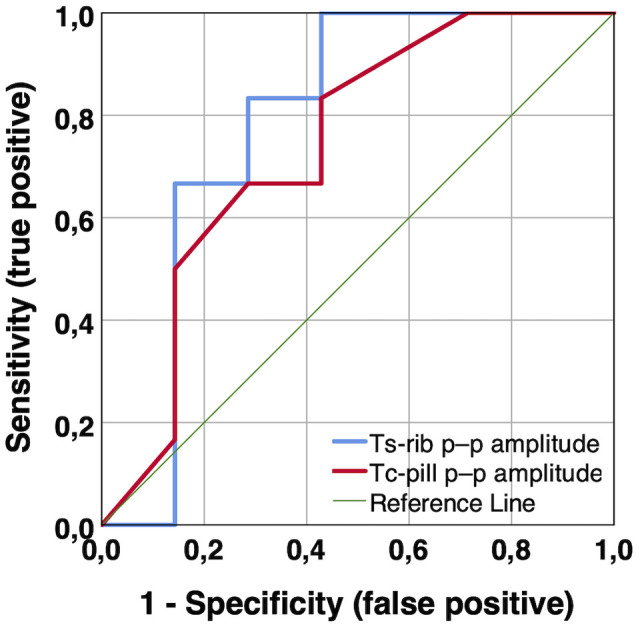
ROC curves of body temperature p–p amplitudes (Ts_rib_ and Tc_pill_) measured during 12-h habitual daily activities to predict MCI. Notes: Ts_rib_ and Tc_pill_ p–p amplitude, AUC = 0.786 (*p* = 0.086) and 0.738 (*p* = 0.153), respectively. AUC, area under curve; MCI, mild cognitive impairment; Tc_pill_, core body temperature from ingestible telemetric temperature pill; Ts_rib_, skin temperature at rib cage; ROC, receiver operating characteristic.

To follow-up on these findings, we computed supplementary receiver operating characteristic (ROC) curves to assess the accuracy of body temperature measures to differentiate healthy individuals from those with MCI. The following accuracy values, i.e., area under the curve (AUC), resulted for the different body temperature measures. Single-point measurements in the laboratory: Ts_rib_ median during sitting and walking, AUC = 0.618 (*p* = 0.089) and 0.626 (*p* = 0.069), respectively; Ts_scapula_ median during sitting and walking: AUC = 0.558 (*p* = 0.402) and 0.551 (*p* = 0.466), respectively; long-term continuous measurements during 12-h habitual daily activities: Ts_rib_, Ts_scapula_, and Tc_pill_ median, AUC = 0.698 (*p* = 0.220), 0.625 (*p* = 0.439), and 0.583 (*p* = 0.606), respectively; and Ts_rib_, Ts_scapula_, and Tc_pill_ p–p amplitude, AUC = 0.786 (*p* = 0.086), 0.762 (*p* = 0.116), and 0.738 (*p* = 0.153), respectively. Exemplary ROC curves for median and p–p amplitude temperature measures are depicted in Figures 7, 8, respectively.

## Discussion

This study aimed to investigate the association of body temperature with cognitive performance in older adults with and without MCI. The two *main findings* were: (1) that better cognitive performance correlates with lower median skin temperature measured in the laboratory (i.e., single-point measurement), as well as with lower median skin and core body temperature measured during 12 h of habitual daily activities (i.e., long-term continuous measurement) and with higher long-term body temperature p–p amplitudes; and (2) that cognitively healthy older adults exhibit lower median skin and core body temperature values, as well as higher body temperature p–p amplitudes compared to their peers with MCI. Interestingly, stronger correlations with cognitive performance were found for the long-term compared to the single-point temperature measurements which potentially highlights an advantage of long-term continuous monitoring approaches compared to single-point measurements.

### Associations of Single-Point and 12-h Median Body Temperature With Cognitive Performance

To our knowledge, this is the first study demonstrating that lower *median* body temperature, assessed either as a single-point measurement in the laboratory or as long-term continuous measurement during habitual daily activities, is associated with better cognitive performance in older adults with and without MCI. These results support our first hypothesis. In the correlation analyses performed with the single-point laboratory body temperature measures, we found statistically significant correlations (*p* < 0.05) of *median* skin temperatures with processing speed (TMT-A). Among the 12-h continuous body temperature measurements, significant correlations of *median* skin and *median* core body temperature were evident with general cognitive performance (QMCI), the executive function inhibition (Stroop), and semantic verbal fluency (category fluency). The finding that the single-point and the long-term body temperature measurements did not correlate with the same cognitive parameters may be related to the differences in the measurement conditions and methods, and participant groups (i.e., whole sample vs. subgroup). The supplementary correlation analyses, performed separately for the two sexes, revealed additional significant associations for *median* skin temperatures with episodic memory (FNAME-12) and phonemic verbal fluency (letter fluency) uniquely in the male subgroup. This could indicate sex differences for the relation of body temperature with cognition, but further research with larger samples of older adults is warranted to substantiate this finding.

We further analyzed if *median* skin and *median* core body temperatures are lower in cognitively healthy older adults compared to older adults with MCI. Here the results showed significant differences among the two groups of older participants for Ts_rib_ measured in the laboratory and additional non-significant differences, which are noteworthy due to moderate to large effect sizes, for Ts_rib_ during the 12-h body temperature measurements. The latter result may imply that the study has lacked statistical power to reach statistical significance (i.e., too few participants in the 12-h measurement subgroup). Supplementary ROC curve analyses revealed that single-point measures of *median* body temperature may not predict MCI with adequate accuracy. Nevertheless, body temperature measures could be combined with other predictors of cognitive performance and integrated into multi-parameter models for the prediction of cognitive decline in older adults.

Similar results were evident for the sitting and walking test conditions in the laboratory, as well as among the two skin temperature measurement sites in both the single-point and long-term measurements. In the long-term measurement, the skin temperatures and core body temperature correlated with different cognitive tests (i.e., Stroop vs. QMCI and letter fluency, respectively). This may be due to methodological and physiological differences in the skin temperature compared to the core body temperature measurements (Taylor et al., 2014).

The observed correlation of cognitive performance with body temperature is coherent with other research findings. Meta-analytic data indicated that patients with AD, where reduced cognitive performance is a hallmark of the disease, exhibit higher core body temperature compared to healthy older adults (Klegeris et al., 2007). Specifically, the rectal core body temperature mesor (i.e., the mean of a cosine function fitted to the 24-h rhythm) was significantly higher in older adults with AD compared to their cognitively healthy counterparts (Harper et al., 2005). Similarly, daytime proximal skin temperature was shown to be higher in AD patients in comparison to non-demented older adults (Most et al., 2012). The cognitive domains and related tests that we assessed in our study were previously shown to mirror the decline of cognitive performance in the transition from healthy to MCI and AD states. Specifically, episodic memory and semantic verbal fluency were suggested to be the most reliable neuropsychological markers of disease progression in MCI/AD (Clark et al., 2009; Drago et al., 2011; Mueller et al., 2015) and predict progression from pre-MCI to MCI/AD (Loewenstein et al., 2012). Furthermore, the FNAME test which reflects episodic memory is related to amyloid deposition in the brain of cognitively healthy persons (Rubino and Andres, 2018). Processing speed, as assessed with TMT-A in our study, is associated with increased brain white matter hyperintensities and reduced fractional anisotropy in older adults (Kuznetsova et al., 2016; Tsapanou et al., 2019). Correspondingly, a recent study proposed that such white matter microstructural deteriorations may provide early biomarkers of AD, since these were already evident in older individuals experiencing subjective cognitive decline (Luo et al., 2019). The subjective cognitive decline represents the earliest preclinical stage of AD, preceding amnestic MCI, with typically preserved performance in memory and/or other cognitive functions when objectively measured (Jessen et al., 2014). Together, these findings may support the assumption that (early) AD-related brain structural changes could be an underlying neurophysiological factor for the association of cognitive performance and body temperature that we found in our study.

### Associations of 12-h Body Temperature Peak-To-Peak Amplitude With Cognitive Performance

We further hypothesized that higher body temperature *amplitudes* during the long-term continuous measurements are related to better cognitive performance. This was confirmed in that we identified statistically significant correlations (*p* < 0.05) for skin and core body temperature *p–p amplitudes* with episodic memory (FNAME-12), processing speed (TMT-A), and phonemic verbal fluency (letter fluency) which represent specific cognitive domains. Additionally, notable non-significant associations with moderate effect sizes for skin and core body temperature *p–p amplitude* with general cognitive performance (QMCI) and executive function (Stroop) were present. Furthermore, body temperature *p–p amplitude* was higher in the healthy compared to the MCI participants, resulting in notable non-significant differences with moderate to large effect sizes for Ts_rib_ (healthy 3.0 ± 1.2°C vs. MCI 1.9 ± 0.4°C), Ts_scapula_ (healthy 3.3 ± 1.1°C vs. MCI 2.4 ± 0.6°C), and Tc_pill_ (healthy 1.5 ± 0.6°C vs. MCI 1.1 ± 0.2°C). These novel findings also comply with previous studies reporting reduced proximal skin and core body temperature amplitudes in dementia patients compared to healthy older adults, as assessed during long-term measurements of the circadian body temperature rhythm (Harper et al., 2005; Most et al., 2012; Leng et al., 2019; Raupach et al., 2019). Supplementary ROC curve analyses suggest that measures of body temperature *p–p amplitude* may predict MCI with better accuracy compared to median body temperatures. Hence, these measures could also be valuable to be integrated with future developments of multi-parameter models for the prediction of cognitive performance in the older population.

Interestingly, the analyses of *p–p amplitudes* showed more results that achieved statistical significance or moderate to large effect sizes for Ts_rib_ compared to Ts_scapula_ (i.e., 4 vs. 2, respectively), which potentially renders the rib cage measurement site more favorable for future applications in wearable monitoring devices. Finally, the correlation analyses with core body temperature *p–p amplitudes* (Tc_pill_) revealed particular additional significant correlations (or moderate to large effects) with Stroop and letter fluency. This observation might again be related to methodological and physiological differences in the skin temperature compared to the core body temperature measurements (Taylor et al., 2014), although circadian rhythms of skin temperature and core body temperature are following a comparable pattern (Most et al., 2012).

As per our results, alterations of circadian rhythms seem to be more pronounced in neurodegenerative diseases (Leng et al., 2019). However, changes in circadian rhythms are also part of the normal aging process where typically the amplitude of many circadian rhythms is attenuated and in some instances, a phase advance of the circadian rhythm is additionally evident (Hood and Amir, 2017). Age-related changes in 24-h circadian rhythms include not only core body temperature rhythms, but also rhythms of waking activity, hormone release (e.g., melatonin and cortisol), fasting plasma glucose levels, and suprachiasmatic nucleus (SCN) firing (Hood and Amir, 2017). The SCN is the master circadian clock and synchronizes rhythmic activities in the body with the cycle of light and dark (Mohawk et al., 2012). Consequently, a diminished downstream output signal from the SCN may additionally affect the strength of rhythms in central and peripheral tissues in aging (Hood and Amir, 2017). The precise mechanism linking circadian rhythm disturbances with neurodegeneration remains unclear, although changes in protein homeostasis, immune and inflammatory processes might be involved (Leng et al., 2019). Nevertheless, behavioral and biological parameters of circadian rhythm disruptions offer interesting targets to monitor neurocognitive long-term health in older adults, since alterations in these biomarkers manifest in preclinical disease states and thus precede the development of AD, AD-related dementias, and other neurodegenerative diseases (Ju et al., 2017; Leng et al., 2018; Musiek et al., 2018).

### Comparison of Single-Point (Laboratory) and Long-term (12-h) Body Temperature Measurements

Our analyses including the long-term continuous body temperature measurements during habitual daily activities produced markedly higher correlation coefficients and effect sizes than the single-point laboratory measurements. An explanation for this outcome could be that the single-point laboratory measurements took place either in the morning or in the afternoon, depending on the participants’ availability. Skin and core body temperatures vary throughout the day, with the nadir normally occurring in the early morning and the peak being reached in the early evening (Czeisler et al., 1999; Most et al., 2012). Hence, the non-standardized time-points of the laboratory measurements presumably generated circadian rhythm-related body temperature variations, which may have superimposed variations related to cognitive performance. This could have led to the observed lower effect sizes in the single-point laboratory measurements. It was not feasible and meaningful to control for the different time-points of measurement in the statistical analyses, since the measurement time-points were widely distributed throughout the day (i.e., from 08.30 to 12.00 AM and from 1.00 to 5.00 PM) and, additionally, interindividual variations in the timing of circadian rhythms are to be expected for example related to activity and light exposure (Czeisler et al., 1999). On the other hand, this outcome may also highlight an increased benefit of long-term (i.e., several hours) over single-point measurements for health monitoring. Within practical settings of remote and continuous health monitoring over several months or years, repeated single-point measurements (preferably measured at consistent time-points during the day) or multi-hour long-term measurements would allow establishing individualized reference ranges. Thereby, the sensitivity to detect disease processes may be raised (Chester and Rudolph, 2011). The continuous long-term body temperature measurements in our study were only performed in a subgroup of the participants, mainly due to limited sensor availability. Moreover, the corresponding procedure of ingesting the telemetric temperature pill for reference data entailed an additional burden for the participants and affected the willingness to participate in the long-term measurements in some cases. Nevertheless, the acquired data provided the required information to evaluate the feasibility of long-term measurements with our prototype system of an ECG chest belt with integrated skin temperature sensors (Figure 1). Therefore, the above-discussed findings and potential advantages of long-term continuous body temperature monitoring warrant future investigations with further developed wearable skin temperature measurement systems.

### Strengths and Limitations

Methodological strengths include the comparably large number of older adults (in relation to previous studies investigating body temperature differences between healthy individuals and patients with MCI or AD), who participated in this initial study to explore direct associations of body temperature and cognition. Nonetheless, future studies would preferably include larger numbers of participants to account for potential fluctuations of body temperature measurements due to intraindividual factors (e.g., physical activity) and ambient conditions. A *post hoc* estimation of statistical power, based on the resulting small to large effect sizes and the number of participants in this study (Faul et al., 2007), revealed that the correlation analyses achieved approximately 37–86% power, whereas the Mann-Whitney tests achieved about 10–22% power. Further strengths comprise the assessment of a variety of cognitive domains and controlling for age in the correlation analyses. The latter seems important since age-related reductions of body temperature (Waalen and Buxbaum, 2011) together with age-related cognitive decline (Salthouse, 2019) might otherwise have confounded the association of body temperature with cognitive performance.

Some limitations have to be considered as well. First, the results from correlation analyses do not automatically imply a causal relationship between the included parameters. Nonetheless, based on the reviewed and presented literature, we conclude that a causal relation of body temperature and cognitive performance parameters is very likely. Second, to ensure the availability of core body temperature data from the ingestible telemetric temperature pill, we have limited the measurement period to 16 h, as transit times through the gastrointestinal tract are highly variable (Bongers et al., 2015). Longer measurement periods (i.e., several days) would offer the opportunity to model and fit circadian rhythms with cosine functions (Harper et al., 2005), which might provide better estimates of average circadian body temperature values, including minimum, maximum, and mesor. Such measurements over several days would require the participants to sequentially ingest several telemetric pills, due to their limited gastrointestinal transit time (i.e., mean 27.4 h, range 4.6–82.8 h; Bongers et al., 2015), and to come by the laboratory several times to read out the data from the pill before its excretion. Therefore, more convenient alternative approaches for long-term measurements, such as the use of non-invasive parameters for the prediction of core body temperature, should be considered to optimize practicality and reduce participant burden. Finally, since a “gold standard” operational definition for diagnosing MCI is lacking and definitions of MCI are highly variable throughout the literature (Jak et al., 2016; Wong et al., 2018), our presented findings related to body temperature differences among healthy and MCI participants is only valid for the applied MCI criteria. Future studies could include brain imaging and blood-based biomarkers to investigate body temperature differences related to MCI caused by different neurodegenerative diseases, such as AD, Parkinson’s disease, and Lewy body dementia.

## Conclusion

To our knowledge, this is the first study exploring the association of body temperature with cognitive performance in older adults with and without MCI. We demonstrated that skin and core body temperature (median and p–p amplitude values), assessed in single-point laboratory measurements and during 12 h of habitual daily activities (i.e., long-term measurement), correlate with both general cognitive performance and with various specific domains of cognitive performance (including episodic memory, verbal fluency, executive function, and processing speed). Moreover, we found that in cognitively healthy older adults, median body temperatures (single-point and long-term) are lower and body temperature p–p amplitudes during continuous long-term measurements are higher than in older adults with MCI. In conclusion, this study highlights the potential of body temperature measures as early biomarkers of cognitive decline, reflecting preclinical and prodromal (i.e., MCI) symptoms of AD. Thereby, the long-term temperature measurements showed greater associations with cognitive performance compared to the single-point measurements. Additionally, long-term p–p amplitude temperature values provided better accuracy to differentiate healthy older individuals from those with MCI than single-point and long-term median temperatures. Based on the presented results, it appears promising to include body temperature sensors into multi-parameter wearable systems for the remote and continuous monitoring of the older population’s neurocognitive health. Future research is warranted to investigate longitudinal changes of body temperature measures in association with normal aging and disease development (e.g., assessed by brain structural or blood-based biomarkers), as well as during therapeutic or preventive interventions (e.g., exercise training) that might affect temperature-related processes in neurodegenerative diseases.

## Data Availability Statement

The raw data supporting the conclusions of this article will be made available by the authors, without undue reservation.

## Ethics Statement

The studies involving human participants were reviewed and approved by Ethikkommission Ostschweiz (EKOS), Switzerland. The patients/participants provided their written informed consent to participate in this study.

## Author Contributions

PE, MB, and SA contributed to the conception and design of the study. PE and MB recruited the participants, conducted and supervised the testing sessions and also performed the statistical analyses. MB executed data processing. PE wrote the first draft of the manuscript. All authors contributed to the article and approved the submitted version.

## Conflict of Interest

The authors declare that the research was conducted in the absence of any commercial or financial relationships that could be construed as a potential conflict of interest.
